# Qualitative behavioural assessment shows individual variation suggestive of affective state changes in early lactation dairy cows

**DOI:** 10.3389/fvets.2026.1744081

**Published:** 2026-06-17

**Authors:** Emily F. Craven, Jorge Alberto Vázquez-Diosdado, Jasmeet Kaler, Alison Russell, Martin J. Green, Jake S. Thompson

**Affiliations:** 1 School of Veterinary Medicine and Science, University of Nottingham, Nottingham, United Kingdom; 2 Agriculture and Horticulture Development Board (AHDB), Coventry, United Kingdom

**Keywords:** affect, dairy cattle, monitoring, positive welfare, QBA, transition cows

## Abstract

Qualitative behavioural assessment (QBA) is described as a ‘whole animal’ approach for evaluating welfare through posture and behaviour to judge their emotional state. Although QBA has been applied in dairy cows for a population-level assessment, its use in evaluating individual cattle remains limited. Given the current limitations in evaluating the affective state in cows, validated methods are needed, including the assessment of QBA, for the assessment at the individual level rather than at the herd level and to assess individual changes over time. In this study, 35 individual cows were assessed both within a group setting and through daily individual QBA immediately post parturition to assess behavioural patterns using principal component analysis (mean 12, range 4–19 days). A sub-sample of 20 cows was clustered according to the observed patterns using Gaussian mixture model-based clustering. A large variation in behaviour was observed among cows on an individual basis, with an increase in principal component 1 (PC1) being associated with a more agitated state, and principal component 2 (PC2) being associated with a more positive and playful/inquisitive state and high arousal states, suggestive of affective state changes. Linear mixed-effects models showed days in milk had a significant effect on both PC1 and PC2, with PC1 decreasing as days in milk (DIM) increased (−0.22, *p* < 0.001) and the opposite for PC2 (0.05, *p* = 0.01). Cows were clustered based on profile patterns of their PC1 and PC2 components, with some cows showing consistent patterns (*n* = 13) and inconsistent patterns of behaviour based upon PC scores (*n* = 7). As manual daily monitoring of cows is unlikely to be practical or feasible, sample frequency analysis was performed, and further analysis showed the potential for less frequent sampling to be able to define these patterns. Our results suggest that individual cow QBA could be useful to identify behavioural changes and pattern profiles after calving, suggestive of an improvement in affective state in housed early lactation dairy cows. QBA at an individual level, compared to group level, could aid welfare assessment, further helping understand dairy cows’ affective states around the post-calving period.

## Introduction

The lack of a negative welfare state does not equal a positive welfare state, and it has been suggested that livestock should be provided with ‘a good life’ (1). The definition of ‘a good life’ has caused much debate, particularly between lay citizens and experts. In general, citizen’s perception has tended to focus on naturalness, whereas experts have emphasised on the animals’ subjective experiences (2). For an animal to have ‘a good life’, assessments need to focus on meaningful measures of positive welfare, in addition to the absence of a negative state, as described in the original Five Freedoms. Positive animal welfare (PAW) is defined with a focus on experiencing positive mental states, going beyond ensuring good physical health and prevention/ alleviation of suffering (3). Consideration should be given to the assessment of positive welfare at both herd and individual levels.

The ability to assess affective state is an important aspect of identifying and facilitating positive welfare because this refers to the mental experience of the animal. Emotion and affective state are considered important in assessing positive welfare because long-term mood states arise from short-term discrete emotions (4). Emotions are important because they guide animal behaviour to make appropriate decisions in response to the environment. Positive states indicate that the animal is coping well within its environment, and negative states may alert to threats, suggesting that affective states are critical for the fitness of the animal (5). Emotions can be interpreted using a dimensional theory, whereby valence expresses positive or negative affective states, and arousal defines high to low (4). Generally (but not always), high arousal is considered to accompany negative emotions, and it has been suggested that spontaneous physiological and behavioural responses typically reflect arousal, whereas learned responses are more valuable for investigating valence (6).

It is not possible to directly measure an animal’s affective state, therefore, behaviour is often used as a proxy measure. This is because an animal’s behaviour and its state—including its health and affective state—are closely coupled and dynamic, both influencing each other over time (7). One specific method that has been developed to quantify an animal’s affective state using behaviour is qualitative behavioural assessment (QBA), and it is the only validated group measure of positive welfare using direct observations in cattle (8). QBA can be used in an integrative approach to describe how animals interact with the environment (9), including capturing fluctuations in behavioural expression, for example, how alert and responsive they are (10), and by judging how the animals feel using visual examination. The focus on behaviour is not what the animal does, rather how it does it (11), knowing that an animal’s body language can reveal important aspects of its mental and physical health and therefore, by extension, its welfare (12). Behavioural change can be measured by observations without intervention to assess responses to stimuli, whereas most physiological changes require interventions.

Originally, QBA was performed using free choice profiling, whereby observers have the freedom to choose descriptive terms for animal behaviour to determine the terms used to describe the behaviours (13). However, as the technique has evolved, fixed descriptor terms are often used, such as in the Welfare Quality Dairy Cattle Assessment Protocol (14). These are designed to incorporate terms associated with affective state, including basic emotions, such as happiness and anxiousness (4), and to reflect both positive and negative arousal and valence (15). Fixed terms ensure that the breadth of behavioural expression is captured without any areas being overlooked by the observer (16). Following observation, analytical methods, such as principal component analysis (PCA), a dimensionality reduction technique based on variance, can be utilised to condense this detailed multidimensional data to a smaller dataset of uncorrelated variables (12).

QBA is often used for a designated purpose, such as farm assurance, and standardised methods are used, such as the Welfare Quality Assessment Protocol for Dairy Cattle (17), for group-level assessments. For the group level assessment, the body language of the whole group is assessed, rather than appraising specific individuals. For research, the QBA methodology used is often adapted to suit the specific requirements and aims of that study. For example, Russell et al. (18) observed that the cows were undisturbed over a period of time, whereas Ebinghaus et al. (19) scored at a single time point to accompany an intervention. Some cattle studies have been undertaken at the individual level and short term, for example, des Roches et al. (20), but most are at a group or population level, such as Aubé et al. (21), Ebinghaus et al. (22), Ellingsen et al. (23), and Russell et al. (18). Individual-level cattle assessments have only been done with a low number of animals and are usually associated with a targeted intervention rather than looking for time-related changes. Group-level assessments may lose some of the sensitivity within the assessment due to substantial differences in individual cow behaviour.

Observation timings are important for QBA implementation and may affect outcomes, as there is potential for QBA variation and is therefore suggestive of affective state changes through the day. In a study of 10 herds, QBA analysis suggested that the three farms exhibited a better ‘mood’ during the late morning compared with the early morning and early afternoon (24). Frequency of observation is also important, as a majority of studies are at a single time point to gain a snapshot in time, and few studies have looked for changes in QBA over a sustained period. A study at the individual level assessed six cows for behavioural changes over an 80-h period following inoculation with *E. coli* (20), based on the hypothesis that behaviour would change as mastitis is a painful condition (25). At the herd level, cows were studied three times a week for 19 weeks following housing interventions (18) and at two time points to assess the effect of housing (26). Although QBA has been validated for use in various contexts in dairy cows to assess affective state (27), the methods are often non-standardised, and it has not been used in individual cows to assess affective state over an extended period.

Given the limitations in the existing evidence base surrounding the use of QBA methodology in individual animals and over an extended period, the primary aim of this study was to assess the validity of using QBA at an individual level in housed, post-partum dairy cows to observe different patterns in behaviour. A secondary aim was to explore the frequency of QBA measurement required to describe and classify a cow’s affective state.

## Materials and methods

Ethical approval was granted by the University of Nottingham School of Veterinary Medicine and Science Committee, number 3917–230824. All methods were performed in accordance with relevant guidelines and regulations, and no direct interventions on the cows were used.

### Animals and housing

The study was conducted at the Centre for Dairy Science Innovation, University of Nottingham, using cows housed all year round. The population group for this study was the freshly calved cow group (study group), which included some sick cows. This group consisted of clinically healthy Holstein cows moved into the group immediately post calving and following removal of the calf for the first few weeks of lactation (parity 1–5, mean 1.8, median). Additionally, some cows in later lactation were kept in the group if they were deemed as ‘sick’ or ‘lame’ at any point in their lactation; however, they were not individually assessed for the study (group mean, 25 cows; max, 40). Freshly calved cows were chosen for this pilot study as it was hypothesised that the combination of parturition, entering lactation, and group changes around this time would enable a greater potential to highlight differences between individuals.

**Figure 1:**
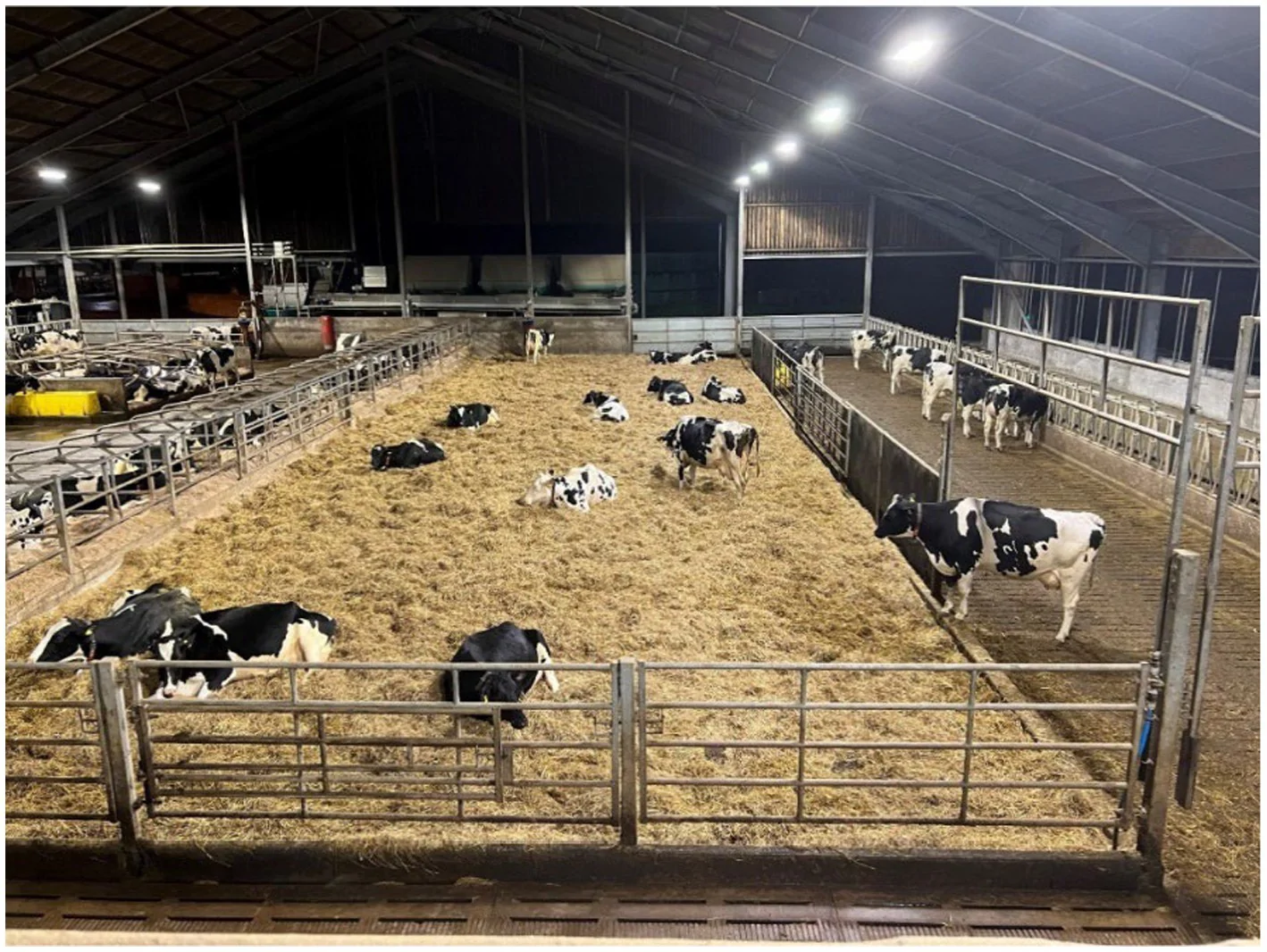
Typical presentation of the cows assessed for QBA. Assessment started in the top left corner and then moved back down the lying space and up the food passage before any cows missed (either because they moved or could not be identified) were observed. The photograph was taken from the viewing platform above the robot console, revealing that, although the cows could technically see the observer, they were not disturbed by them. If any signs of disturbance were seen, then the cows were allowed time to habituate.

The study group pen consisted of a straw yard bedded area (36 × 11.6 m), with slatted passageways (5.3 × 45 m) along the feed face (45 m) and around a single Lely Astronaut A4 robot for automatic milking (Figure 1). Additionally, cows had access to an automatic rotary brush and two water troughs. Partial mixed ration (PMR) was fed in the morning at 0900 h and regularly pushed up throughout the day. Additional concentrate feed was provided via the robot during milking. The feed passage was scraped each morning.

Freshly calved cows or sick cows were added to the group each morning, while other cows were removed to the cubicle yards. The criteria for removal were >14 days post calving, milking consistently, and the stockperson being satisfied with health and performance. On removal, if cows had been recruited, then they exited the trial. Management tasks such as group changes, feeding up, and scraping out generally occurred between 0700 h and 0900 h. Other irregular disturbances occurred due to management tasks such as treatments, group changes, or collection for milking. Cows were managed in line with the commercial care and management procedures for the unit, with no interventions for the trial.

### Study recruitment

Given that the herd operated an all year-round calving system, for the individual QBAs, cows were recruited either at the point of being moved into the fresh yard (day 0 at calving) or, at commencement of the trial, if they were in the first 7 days of lactation (full recruitment schedule given in Supplementary Table 1). Recruitment finished on day 14 of the trial to ensure that each cow was assessed for a minimum of 7 days. Observations only took place in the freshly calved group, indicating that cows were observed for the whole duration of the trial period unless they exited the trial pen for management purposes. A convenience sample of 35 cows was achieved based on the total number of calvings during the observation period. For reference, simulated data from clustering methods indicate that using a minimum of 10 repeated samples, with high separation between 2 clusters, and a minimum number of 30 cows would be required for a 95% Bayesian information criterion (BIC) detection (28).

### Qualitative behavioural assessment

QBA was performed daily at 1900 h for 21 consecutive days between 18 November 2023 and 8 December 2023. The time was chosen to avoid human disturbance from routine management tasks such as group changes, routine vet and foot trimming visits, and routine farm management interference (feeding and cleaning). It was assumed that this time point was therefore representative of the group’s undisturbed behaviour.

Assessments were then performed at the group and individual level using QBA based on the Welfare Quality assessment protocol for the group analysis and adapted for individual use (29). The whole group (fresh and sick cows) was initially assessed for 20 min before the recruited individuals (fresh cows only) were assessed, each for 2 min. One trained observer performed all the observations (EC: female, aged 34, veterinarian, experienced with dairy cows). All observations were performed at a single vantage point on the viewing platform above the robot console (shown in Figure 1). The whole group could be appraised from this point, and the cows remained undisturbed. If the cows appeared to be disturbed by the observer’s presence, they were allowed several minutes to habituate before observation recommenced.

Twenty descriptors were scored on a visual analogue scale (VAS), in which a minimum point indicated that the expressive quality indicated by the term was absent and the maximum point meant that it was dominant. Negative terms indicated that a higher score was more negative, and positive terms indicated that a higher score was more positive. The terms used were *active, relaxed, fearful, agitated, calm, content, indifferent, frustrated, friendly, bored, playful, positively occupied, lively, inquisitive, irritable, uneasy, sociable, apathetic, happy, and distressed.*

A score was given for each term by manually drawing a line on the assessment sheet on the visual analogue scale that best represented the level of the behaviour relative to the descriptive attribute seen. The line was then manually measured from the zero minimum point to the plotted assessment line, generating a score of between 0 and 125 for each attribute. Further explanation of the VAS scoring system is provided by the Welfare Quality Network protocols (29).

Cows were assessed by scanning from the top left corner of the pen and working systematically around the group, observing the whole lying area before moving back up the feed face to finish in the top right corner (Figure 1). Given that the individual observation period took approximately 1 h and cows did move around the pen, some cows were missed. Any cows missed following this protocol were identified by rescanning the pen for their individual ID and assessed at the end of the observation. Binoculars were used to ensure the quality of observation and to aid identification of cows (using ear tags, freeze brands, or neck collars). This systematic approach was to ensure baseline behaviour was assessed rather than the observer identifying cows due to their behaviour.

### Statistical analyses

The data were analysed in Microsoft Office Excel and RStudio version 4.4.1 (30). Additional packages used in RStudio were *ggcorrplot* (31), *factoextra* (32), *ggplot2* (33), *dplyr* (34), and *tidyverse* (35).

#### Descriptive information

Basic cow information, such as identification, parity, and calving date, was taken from the farm management software Uniform-Agri and Lely Horizon.

#### Group and individual-level PCA analysis

Analysis was conducted for the assessment at both group and individual levels (n = 35 recruited cows), separately. For both, the raw QBA descriptor linear measurements, at both group and individual levels, were manually measured and normalised using the scale function (achieved by dividing the (centred) columns of x by their standard deviation), before being further analysed using a Principal Component Analysis (PCA) to reduce the number of variables (18, 36). Descriptive assessment of QBA was conducted graphically for both the group and individual level assessments to facilitate visualisation of the important variables contributing to the key principal components. The first two principal components (PC1 and PC2), which explained the highest percentage of the variation and had eigenvalues>1.0, were retained for additional inferential analysis in accordance with standard procedure (37).

#### Linear mixed effects model

A linear mixed effects model was constructed to test the effect of parity and days in milk on the principal component scores (38). For the model, cow ID and date were included as random effects, and days in milk and parity (categorical: multi- or primiparous) were fixed effects. Explanatory variables were retained in the models when the *p*-value was < 0.05. A separate model was built for outcome variables PC1 and PC2.

The final model took the following form:

Y_ijk_ = *α* + β1 X1_ijk_ + β2 X2_ijk_ + U_j_ + R_k_ + e_ijk_,

Uj ~ N (0, σ^2^_u_).

Rk ~ N (0, σ^2^_r_).

e_ijk_ ~ N (0, σ^2^_e_),

where subscripts *i* and *j* denote the *i*th observation of the *j*th cow for the kth date, respectively. *Yijk* is the principal component for the *i*th observation of the *j*th cow on date k, α is the model intercept, X1 is the covariate days in milk, X2 is a categorical variable to represent primiparous versus multiparous cows, β1-2 are respective fixed effect coefficients, U_j_ is a random effect to account for CowID, Rk is a random effect of date (both assumed to be normally distributed with mean = 0 and variances = σ^2^_u_ and σ^2^_r,_ respectively), and e_ijk_ is the residual model error (with mean = 0 and variance = σ^2^_e_). Residual plots and Q-Q plots were used to visually check that the model met the homoscedasticity and normality assumptions of linear regression (39).

Days in milk (DIM) was also tested with polynomial terms (two to four inclusive) and with interactions; however, the model performance was not improved; hence, the simplest model with good performance was retained.

#### Cluster analysis

Individual cow similarity and variability in QBA scores were explored by comparing plots of principal component scores with days in milk and then undertaking cluster analysis of the data.

Clustering was performed in RStudio using the first two principal components and the *mcclust* package to process an MCMC sample of clusterings (40). This approach uses methods that find a single best clustering to represent the sample, which are based on the posterior similarity matrix or a relabelling algorithm (41). A subsample of cows that had a complete dataset between 0 and 10 days in lactation observations was included in this analysis (*n* = 20). Any cow with fewer than 10 observations was excluded. Cows were allocated to a cluster based upon the maximum posterior probability (42).

#### Sample frequency analysis

To explore the ability to cluster cows based on less frequent data, MclustDA, using eigenvalue decomposition discriminant analysis (EDDA), was used to train a discriminant analysis-based model using the cluster observed from the original clustering method above (43). This model was then used to classify based on sub-samples of each cow’s data, which was undertaken by repeat sampling without replacement of each cow’s principal component data 100 times between 1 and 10 days in milk datapoints (total = 900 runs per cow), with the additional rule that day 0 was always included. These were arranged in order of days in milk and reanalysed using the MclustDA model. Due to the sample frequency for each pattern, the cluster probability output was combined for clusters 1, 3, and 4 (inconsistent patterns in PC score) and compared with the consistent pattern cohort (cluster 2). If the combined probability for being in cluster 1,3, and 4 was greater than 0.5, the cow was assigned to a ‘mixed inconsistent’ cluster as opposed to the reference of ‘consistent cluster 2’. Confusion matrices were used to compare the accuracy of the new subsample cluster identification based on sample frequency.

## Results

### Totals

Thirty-five cows (18 multiparous and 17 primiparous) were recruited for individual analysis and were assessed for a mean of 12.2 days (median 13 days, range 4–19 days). Twenty-six cows were recruited on day 0. The mean milk yield for the recruited cows during the study period was 25.5 L (median 24.2 L, range 10.81–43.42 L).

### QBAs

#### Group-level PCA

**Figure 2:**
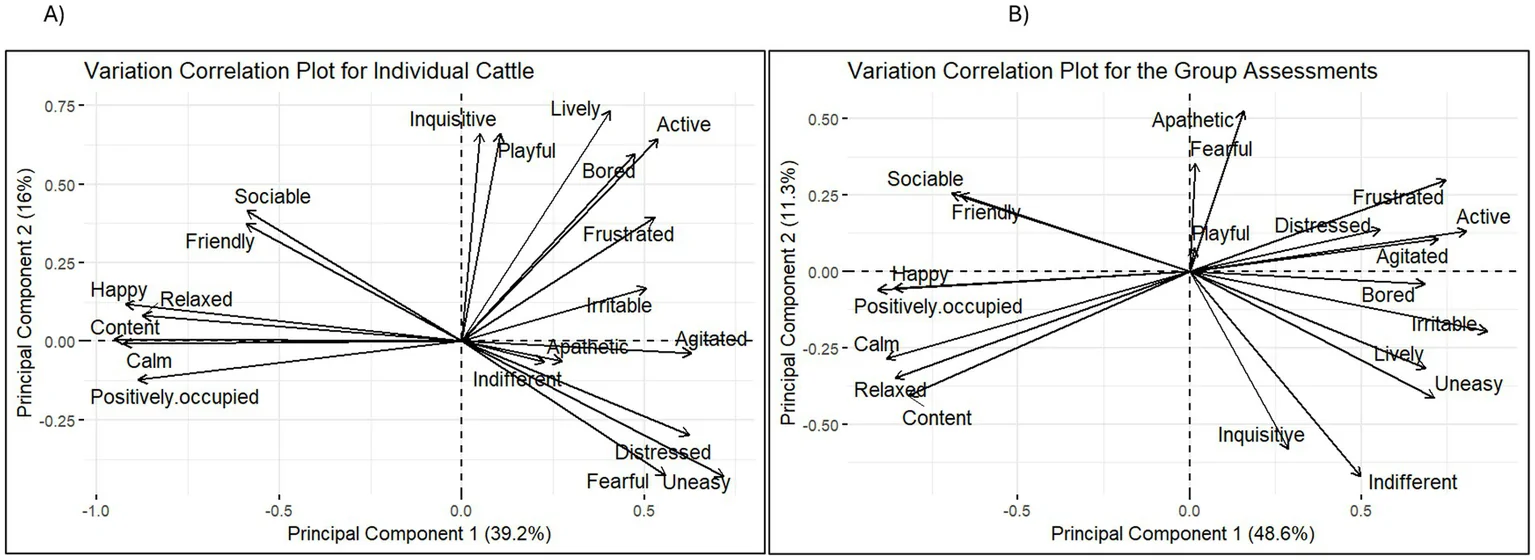
Variation correlation plot displaying the relationship between all variables in PC1 and PC2. **(A)** PC1 was negatively associated with ‘positively occupied’ and ‘happy’ and positively associated with terms such as ‘frustrated’, ‘active’, and ‘irritable’. PC2 was positively associated with ‘apathetic’ and ‘fearful’ and negatively associated with ‘inquisitive’ and ‘indifferent’. **(B)** PC1 was negatively associated with ‘positively occupied’ and ‘happy’, ‘calm’, ‘content,’ and ‘relaxed’ and positively associated with terms such as ‘frustrated’, ‘agitated,’ and ‘irritable’. PC2 was positively associated with ‘inquisitive’ and ‘playful’.

PCA of the group QBA scores identified five principal components with eigenvalues> 1. However, the first two principal components (PC1 and PC2) explained a substantial proportion of the variance in the data (59.9%) and were therefore retained for analysis, with PC1 contributing to 48.6% of the variance. PC1 was negatively associated with ‘positively occupied’ and ‘happy’ and positively associated with terms such as ‘frustrated’, ‘active’, and ‘irritable’. PC2 was positively associated with ‘apathetic’ and ‘fearful’ and negatively associated with ‘inquisitive’ and ‘indifferent’. Table 1 shows the full list of terms with both components and associated loading value, and Figure 2A displays the relationship between the variables.

**Table 1:** Group and individual analysis: principal components 1 and 2 and associated loading value for each behavioural descriptive term.

Descriptor	Group level		Individual level	
	PC1	PC2	PC1	PC2
Active	**0.26**	0.09	0.19	0.36
Relaxed	−0.28	−0.24	−0.31	0.05
Fearful	0.01	**0.24**	0.20	**−0.24**
Agitated	0.24	0.07	**0.22**	−0.02
Calm	**−0.29**	−0.19	**−0.33**	−0.00
Content	−0.27	−0.28	**−0.34**	0.00
Indifferent	0.16	**−0.46**	0.08	−0.04
Frustrated	0.25	0.20	0.19	0.22
Friendly	−0.22	0.17	−0.21	0.21
Bored	0.22	−0.03	0.17	0.33
Playful	0.01	0.05	0.04	0.37
Positively Occupied	**−0.30**	−0.04	−0.32	−0.07
Lively	0.23	−0.22	0.15	**0.41**
Inquisitive	0.09	**−0.40**	0.02	**0.37**
Irritable	**0.28**	−0.13	0.18	0.09
Uneasy	0.23	−0.28	**0.26**	**−0.24**
Sociable	−0.23	0.17	−0.21	0.23
Apathetic	0.05	**0.36**	0.10	−0.04
Happy	−0.28	0.04	−0.33	0.07
Distressed	0.18	0.09	0.22	−0.17

Positive terms are in white, negative terms are in grey, and the two most positive and two most negative contributing terms are in bold.

#### Individual level PCA

PCA of the individual QBA scores identified 4 principal components with eigenvalues > 1. However, the first two principal components (PC1 and PC2) explained much of the variance in the data (56.2%) and were therefore retained for analysis, with PC1 contributing 39.2% of the variance. PC1 was negatively associated with ‘positively occupied’ and ‘happy’, ‘calm’, ‘content’, and ‘relaxed’, and positively associated with terms such as ‘frustrated’, ‘agitated’, and ‘irritable’. PC2 was positively associated with ‘inquisitive’ and ‘playful’. Table 1 shows the full list of terms with both components and associated loading value, and Figure 2B displays the relationship between the variables.

#### Individual variability

**Figure 3:**
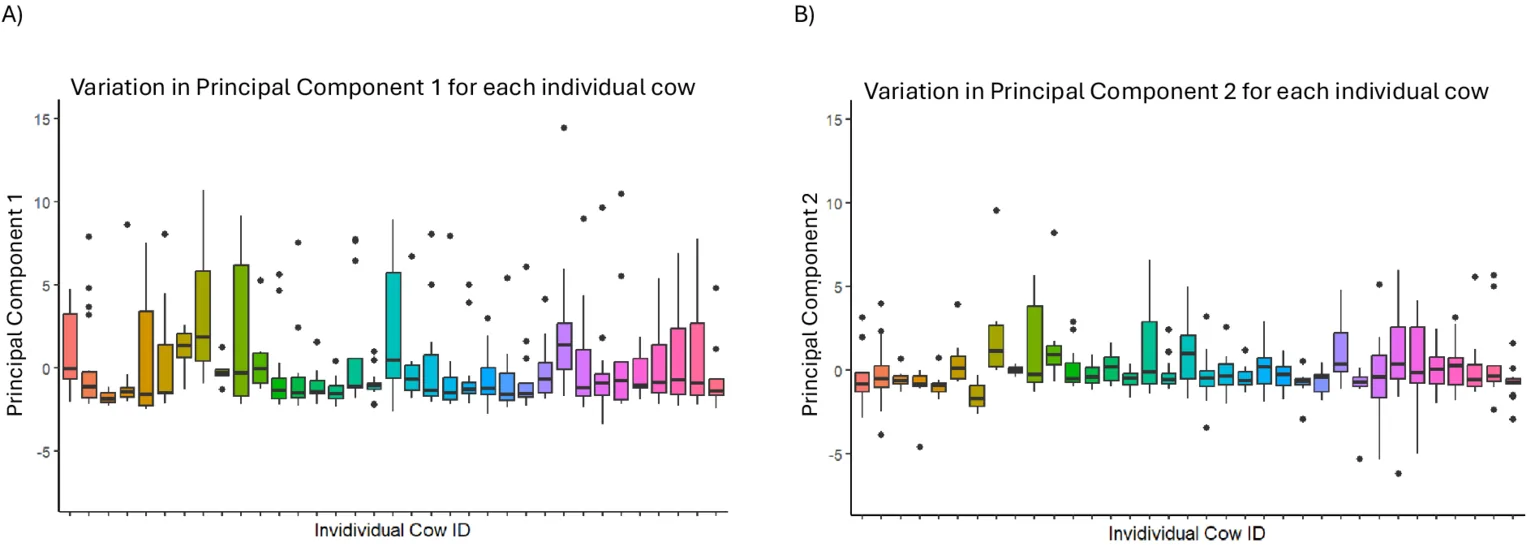
Plot showing the degree of variation in PC scores for each individual cow throughout their observation period. **(A)** shows the degree of variation in PC1 (mean interquartile range 2.28, median range 8.54, range of ranges 1.13–16.2) and **(B)** shows the degree of variation in PC2 (mean interquartile range 1.45, median range 4.61, range of ranges 0.82–12.2).

There was a large variation across individual cows and the extent of variability seen is described by the range of ranges (PC1 1.13–16.2; and PC2 0.82–12.2). Visual assessment showed that some cows appeared to have greater variation in their PCA scores than others during the observation period (Figure 3).

#### Linear mixed-effects modelling

**Figure 4:**
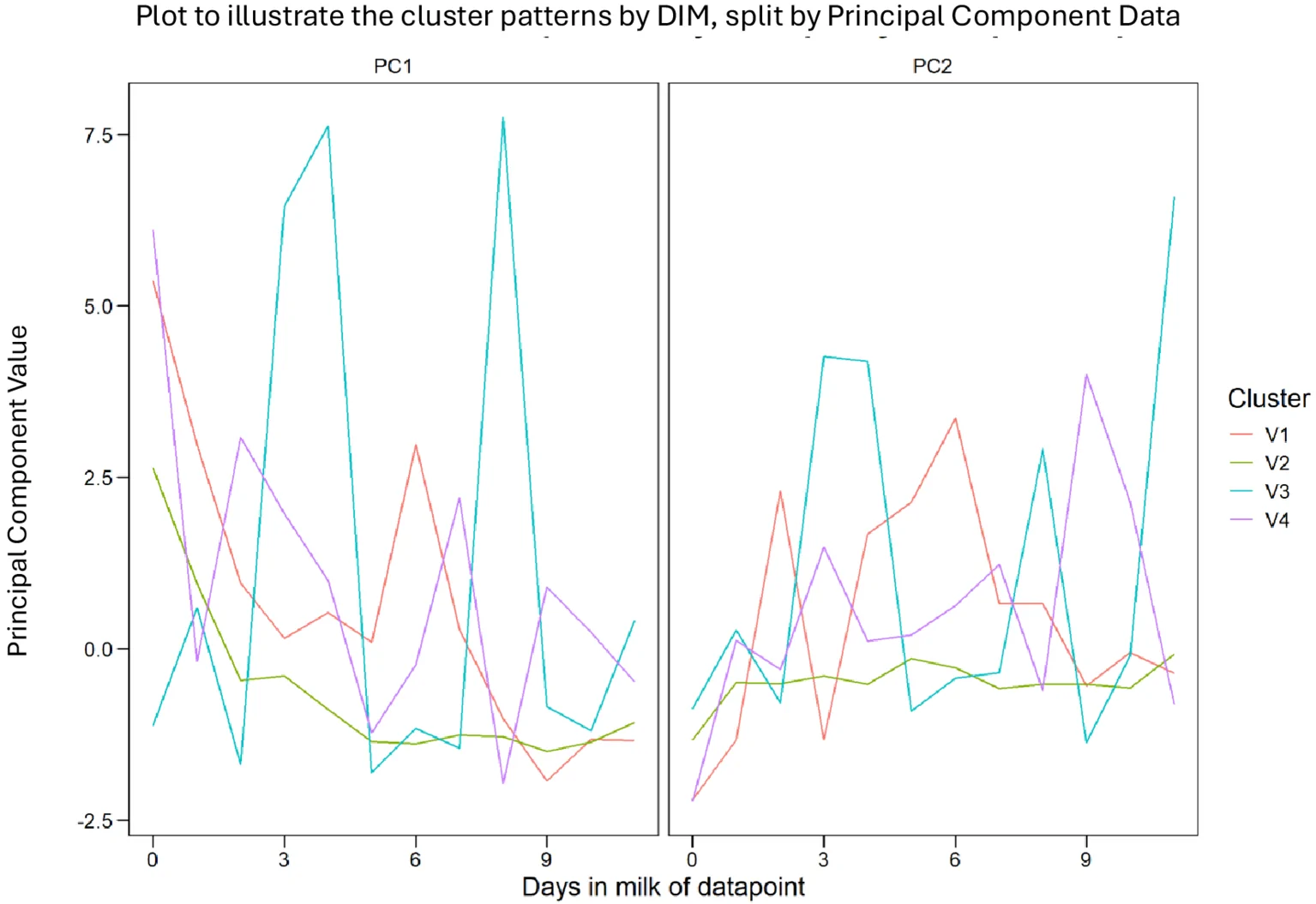
Line plot showing the days in milk (DIM) pattern of PC1 (left side graph) and PC2 (right side graph) for the four clusters identified. V1 shows general variation, V2 shows more stability, and V4 shows a marked change initially and then more stability. V3 represents one cow that did not fit into any of the other clusters, showing marked variation.

Linear mixed-effects models were used to assess for statistical significance, and the results are presented in Table 2. For individual cows, days in milk had a significant effect on both PC1 and PC2 scores, revealing that affective state improved as days in milk increased (as shown by a negative estimate for PC1 and a positive estimate for PC2; Figure 4).

**Table 2:** Results of the linear mixed effects modelling assessing PC1 scores with parity and days in milk.

Principal component	Coefficients of the model	Estimate	Standard error	*p*-value
PC1	Intercept	1.57	0.39	
	Days in milk	−0.22	0.03	<0.001***
	Cow vs. heifer	0.31	0.29	0.31
PC2	Intercept	−0.21	0.22	
	Days in milk	0.05	0.02	0.01*
	Cow vs. heifer	−0.23	0.26	0.38

Days in milk had a statistically significant effect on PC1 score (*** *p* < 0.001) and PC2 score (* *p* < 0.01). All data were included in this analysis (35 cows, 426 observations).

#### Exploration of individual-level variation

**Figure 5:**
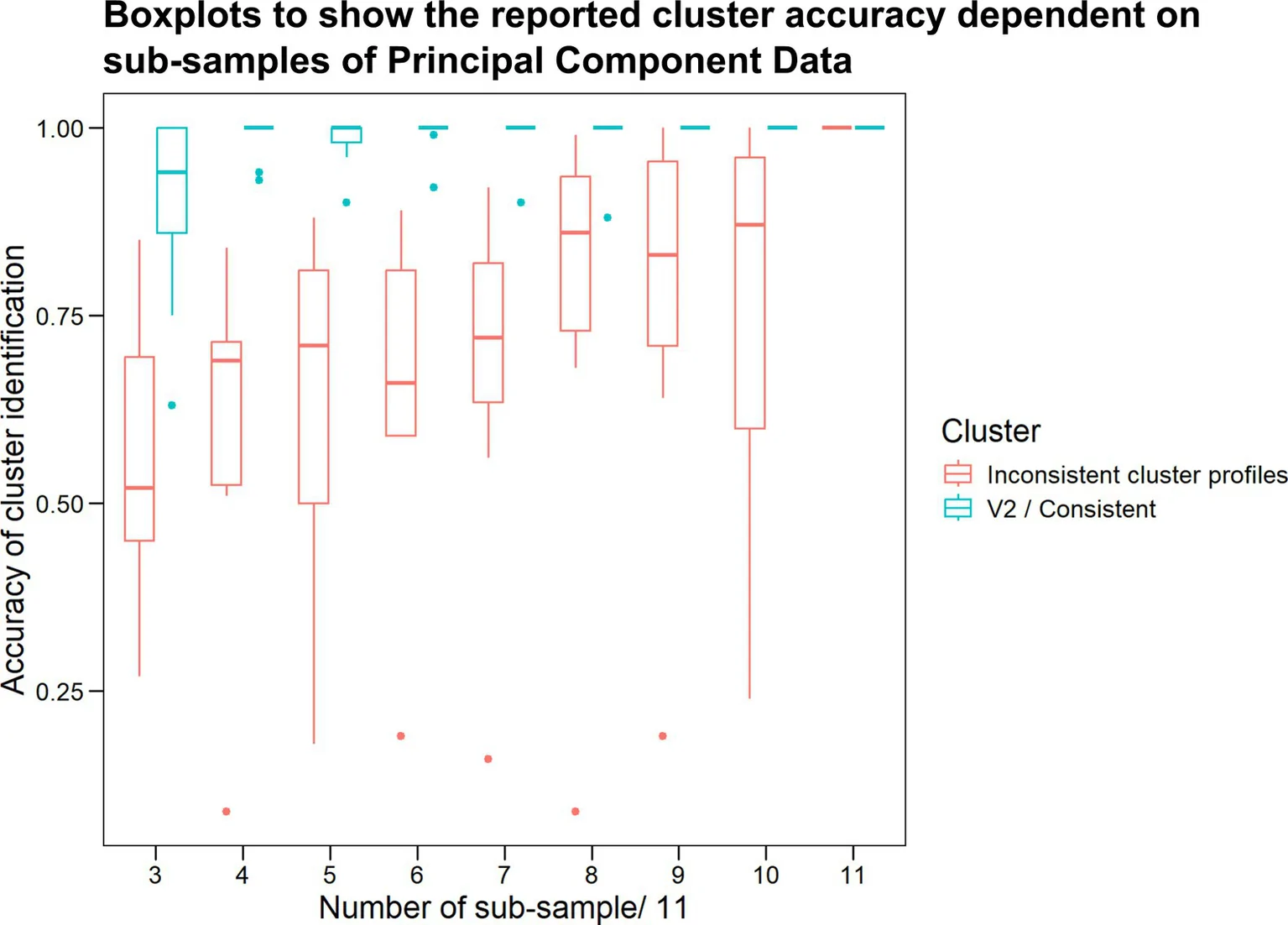
Plot showing the calculated cluster accuracy dependent on sub-samples of PC data using day 0, plus 1–10 datapoint sub-samples out of 11. Cluster 1, 3, and 4 combined into a mixed group (red, *n* = 7). Cluster 2 is in blue (*n* = 13).

The variation shown in Figure 5 ranged from minimal variation in principal component score over the observation period (the consistent cows) to an effect with DIM. This DIM effect was either variable over the entire trial period (the inconsistent cows) or more commonly showed diminishing variation over the observation period (the tailing off cows) and was evaluated using cluster analysis.

#### Clustering

Twenty cows were eligible for cluster analysis based on having been a complete data set for the first 10 days in milk. The 20 cows were categorised into four clusters (V1-4) based on model BIC, as illustrated. The clusters contained 3, 13, 1, and 3 cows, respectively. Cluster 2 (V2) contained a majority of cows and represented the relatively consistent group, whereas cluster 1 (V1) and 4 (V4) represented the groups with more variation. Cluster 3 (V3) represents one cow that did not fit into any of the other clusters but showed marked variation. The effects were more marked for PC1; however, the same patterns can be noted for PC2 (Figure 4).

#### Sample frequency analysis

Given the individual variation seen in cows and the patterns identified with the clustering, sample frequency analysis was undertaken to see how frequently individual assessment would need to be performed in order to classify these patterns accurately between consistent (V2) and inconsistent (V1, V3, V4) cluster patterns. Results are illustrated in Figure 5. As more samples were taken, the accuracy of the model increased, but with the more reliable sampling frequency for accuracy occurring above five sample days in 11 for cows with a variable PC pattern [V1, V3, and V4, (*n* = 7)]. The single cow in cluster 3 always performs variably, dependent on subsample, indicating potential for over-fitting or under-representation in the data (*n* = 1). The model performs well for all cows irrespective of subsampling when cows have a more linear PC pattern (V2, *n* = 13), as shown by the small interquartile range. More variability is seen for the combined V1, V2, and V4 group, as shown by larger interquartile ranges.

## Discussion

The study results suggest QBA changes in the immediate post-partum period in agreement with (44) a move towards more positive elements as days pass since calving. Cows appear to fall into two main groups: those that are consistent (and generally positive QBA) and those that are more inconsistent. Given that QBA is used as a proxy measure of affective state, this study suggests the potential for individual-level QBA to be used as an indicator for observable differences in emotion and therefore affective state. Patterns of PC1 and PC2 showed a statistically significant effect of change dependent on days in milk. Since an animal’s affective state responds rapidly to its physiological state and events in the external environment (4), it follows the information on behaviour and emotion would empower farmers to respond more rapidly and effectively to protect welfare and mitigate losses in productivity and quality caused by disease and distress post a calving event (45).

Although changes based on QBA have not previously been demonstrated post-calving, it is consistent with previous findings using alternative approaches. Changes to performance and behaviour post calving following NSAID administration have been shown even following non-assisted calvings, suggesting that calving is associated with pain and affective state changes (46). Pain has been associated with a combination of elements, such as an aversive sensory stimulus and emotional experience (47). Therefore, this research may add further detail regarding the potentially emotional responses around parturition. While calving is painful and potentially influences affective state changes, with changes in PC1 and PC2 being statistically associated with days in milk, it should be noted that other factors contributing to negative affective states, associated with the removal of the calf, may also be involved (48).

The PCAs observed at both group and individual levels showed that positive and negative emotions were generally differentiated and reflected in different quadrants, as proposed by Forkman and Keeling (15), although the high and low arousal states were less clearly demarcated. Group and individual PCAs showed similar patterns and contributions to the dimensions, which provides evidence for the use of QBA in individual cattle, given that much of the validation work has been performed at the population level. Although previous studies have occasionally used QBA at an individual level, performance has not been compared with group-level analysis or to capture baseline behaviour. For example, Boyer des Roches et al. (25) used it to capture acute disease processes, comparing the change in QBA score when affected by mastitis rather than comparing individual cow QBA scores as a proxy for affective state variation among individuals. This study enables variation among individuals to be compared and describes different profile patterns in cows, as highlighted by the clustering analysis.

In this study, the descriptive terms generally contributed similarly to the dimensions such as ‘content’, ‘positively occupied’, and ‘calm’. However, a few terms, such as ‘irritable’ and ‘fearfulness’, showed marked changes in contribution between the group and individual level assessments and may be a reflection that the fixed terms in Welfare Quality were developed for population-level assessments. The contribution of these specific terms is likely diluted in group assessments due to the granularity of conducting QBA at the individual level. Certain attributes, such as fear, are likely to be very differently represented by different individuals owing to differences in expression, for example, stoic cows. It would appear plausible, for example, that ‘fearfulness’ has a higher contribution at the individual level, given that it was shown to be more associated with first lactation, particularly around calving and in the first few days of lactation, as many of the stressors encountered are novel events, and novelty is a fear stressor (49). Increased fearfulness would therefore be clearly observed during an individual-level assessment, particularly in the first few days of lactation. However, this behaviour may be less evident when assessing group-level dynamics, particularly in an all-year-round calving herd, such that all cows within a group being appraised are at different lactation stages.

The effect of day-to-day variability due to on-farm events could have also contributed to some of the variability in QBA scores, as mood can vary through the day (24). However, some variation would continue to exist compared to other studies due to the dynamic nature of movements in and out of the group, particularly first-lactation animals, given the known behavioural effects of regrouping dairy cattle (50). For example, from previous literature, when QBA is used in Welfare Assessments, it is usually used in stable social groups at all points of lactation so that a single measurement can be used as a proxy baseline for the unit (14, 26, 29). This can be achieved by observing at multiple points so that the whole group can be observed.

Attempts were made to mitigate the effects of management and group changes by assessing at 1900 h when normal management interventions with the cows had ceased, and group changes were done at 0800 h; however, there may still have been a residual effect, given that von Keyserlingk et al. (50) found an effect for hours and days post regrouping. The acute behavioural effects of regrouping dairy cows (50) may have had a more profound effect on the individuals assessed than the group combined. It is important to further explore a parity-related effect on observed QBA, given that the effect size in this study was not statistically significant with the linear mixed model. However, this may reflect the small sample size, since previous studies have that first-lactation cows experience greater stress (49, 50).

Although there was a general improvement in emotion observed and less variation by the days in milk effect, there was much greater variation among some of the individual cows. It has been recognised that cattle have different temperaments and that these are often heritable and influence production and welfare (51). By using QBA to demonstrate the potential changes in affective state and variation of how the different cattle respond to the calving period, early insights into individual’s inability to cope may be determined, which could aid in management, performance, and ultimately welfare. Furthermore, the variation observed may offer insight into a cow’s potential for resilience or future performance. The influences of genetics, environment, behaviour, and physiology will impact individual cow welfare, and correspondingly, in the feedback loop, this will directly influence subsequent behaviour and physiology (52).

The sample frequency analysis have suggested that, while daily QBA is optimal during the first 10 days of lactation, there may be scope to reduce the number of observations and still be able to determine the individual variability patterns seen in the clustering analysis. The analysis suggests that the model still performs adequately when the cows are sampled less frequently, particularly for the cluster 2 cows that show less variation. Given that this substantially increases the feasibility of using QBA at the individual level, further investigation over a longer time period and with a larger sample size. The model suggests a minimum of five samples, including day 0, provide acceptable performance (72% for the median cow in the inconsistent clusters and 100% for the median cow in the consistent clusters). Whether a cow was classified within a variable or consistent cluster, these findings suggest that monitoring every 48 h or intensively for 5 days rather than daily throughout the period should be effective. With a larger sample size to train the model, the performance of the model could be improved, and less frequent sampling may be required. A limitation of the study was the difficulty of cow identification when they were lying, meaning that they were scored out of sequence. This may have increased the likelihood of cows being identified when exhibiting interesting behaviours and moving around more frequently, potentially influencing QBA terms such as the activity score. This is known as anchoring bias, whereby a single gesture might alert the observer to score the cow and skew the assessment [(53) for more information on the anchoring effect]. Anchoring bias should have been minimised by identifying cattle systematically and then assessing for the full 2-min period before turning away to make the assessment, as described by the protocol.

Other limitations of this study include the fact that it was a small dataset using adult, housed, high-yielding Holstein female cattle immediately post-calving. Although the results are indicative of a generalisable effect, further studies on a larger dataset and at different points in lactation are required, indicating that infrequent but important expressions of terms were not present and have not been accounted for. Using PCA, some variation was omitted by only using the two most significant components PC1 and PC2, which explained 55% of the variation (39.2% PC1 and 16% PC2). Particularly given the small sample size, this suggests that more subtle effects represented within the remaining 45% of the variation from other terms may not have been identified and thus included in the model classification analysis.

Although considered a validated approach (18), the integrity of QBA is sometimes doubted as it can be considered subjective and anthropomorphic. Objectivity is usually shown using intraobserver and interobserver reliability, which were not performed in this study. Interobserver reliability was not deemed necessary because only one observer was involved in the study, and the analysis was focused on changes and trends within the observation rather than absolute values. Intraobserver reliability was not performed due to the short-term nature of the study. The usual described method for performing IOR is to watch the same video at repeated time points and compare the values using a correlation ranking analysis such as Spearman’s rank or ICC. By performing this only a few weeks apart, it is likely that the observer would have remembered the videos, and this would affect validity. The fact that the cows were at different assessment days and the days in milk effect was clear and statistically significant suggests that it was an actual observed effect rather than affected by changing across the days.

## Conclusion

This study shows that QBA can be used to describe emotional state patterns at an individual level in housed early lactation dairy cows. A large variation has been observed among cows on an individual basis, with an increase in PC1 being associated with a more agitated state, and PC2 being associated with a more positive and playful/inquisitive state. Cows were also grouped into clusters based on their early lactation profiles, with some cows showing seemingly little variation and others showing appreciably more inconsistency. This links into current thinking regarding early lactation effects on resilience and subsequent prediction of future health, welfare, and performance, and presents a novel application of how QBA may be utilised in practice to interpret how cows are coping within their environment on an individual level. The utilisation of this could aid in positive evidence-based change in management practices. Further study could help diagnose whether QBA at the individual cow level would assist in early detection of disease or discomfort when a negative change to affective state is identified.

## Data Availability

The raw data supporting the conclusions of this article will be made available by the authors, without undue reservation.
